# Lack of Evidence for Vasoactive and Inflammatory Mediators in the Promotion of Macular Edema Associated with Epiretinal Membranes

**DOI:** 10.1038/s41598-017-08997-6

**Published:** 2017-09-06

**Authors:** Brooks P. Applewhite, Savalan Babapoor-Farrokhran, David Poon, Syed Junaid Hassan, Elizabeth Wellmann, Howard S. Ying, Gregg L. Semenza, Silvia Montaner, Akrit Sodhi

**Affiliations:** 1 Wilmer Eye Institute, Johns Hopkins University School of Medicine, Baltimore, MD 21287 USA; 2 Department of Ophthalmology, Boston University School of Medicine, Boston, MA 02346 USA; 3 Department of Pediatrics, Johns Hopkins University School of Medicine, Baltimore, Maryland United States; 4 Department of Medicine, Johns Hopkins University School of Medicine, Baltimore, Maryland, United States; 5 Department of Oncology, Johns Hopkins University School of Medicine, Baltimore, Maryland United States; 6 Department of Radiation Oncology, Johns Hopkins University School of Medicine, Baltimore, Maryland United States; 7 Department of Biological Chemistry, Johns Hopkins University School of Medicine, Baltimore, Maryland United States; 8 McKusick-Nathans Institute of Genetic Medicine, Johns Hopkins University School of Medicine, Baltimore, Maryland United States; 9 Department of Oncology and Diagnostic Sciences, School of Dentistry, University of Maryland, Baltimore, Maryland United States; 10 Department of Pathology, School of Medicine, University of Maryland, Baltimore, Maryland, United States; 11 Greenebaum Cancer Center, University of Maryland, Baltimore, Maryland, United States

## Abstract

The development of symptoms in patients with epiretinal membranes (ERMs) often corresponds with the accumulation of interstitial fluid in the retina [i.e., the development of macular edema, (ME)]. To explore the potential value of pharmacologic therapeutic options to treat ME in patients with ERMs, we examine here the expression of vasoactive and inflammatory mediators in the vitreous of patients with idiopathic ERMs. We observed that vitreous concentrations of classic vasoactive factors (e.g., vascular endothelial growth factor) were similar in ERM patients with ME compared to controls. Using an array assessing the expression of 102 inflammatory cytokines we similarly did not observe a marked difference in cytokine expression in the vitreous of most ERM patients with ME compared to control patients. While the array data did implicate a group of inflammatory cytokines that were elevated in a subset of ERM patients who had severe ME (central subfield thickness ≥450 μm on spectral domain optical coherence tomography), expression of 3 of these inflammatory cytokines, all previously implicated in the promotion of ME in ischemic retinal disease, were not elevated by quantitative enzyme-linked immunosorbent assay. We conclude that therapies modulating vasoactive mediators or inflammatory cytokines may not affect ME in ERM patients.

## Introduction

Epiretinal membranes (ERMs) are common in the elderly population, with peak prevalence (up to 1/3 of patients) between age 70 and 79^1^. Although they may be associated with other ocular conditions, most ERMs are idiopathic^2^. 10% of idiopathic ERMs progress from grade 0 (asymptomatic cellophane maculopathy) to grade 2 (pre-retinal macular fibrosis) over 5 years^3, 4^. Approximately 80% of patients with grade 2 ERMs will have symptoms, including blurred vision, metamorphopsia, monocular diplopia, and micro/macropsia^5^. These symptoms often correspond with the accumulation of interstitial fluid in the retina (i.e., the development of macular edema). While not formally tested, it is nonetheless hypothesized that macular edema (ME) in ERM patients may be a consequence of microvascular injury caused by mechanical traction on the retinal vasculature by the membrane^6^. Accordingly, standard treatment of symptomatic ERMs with ME is vitrectomy with surgical excision of the membrane.

Histological studies of surgically removed ERMs have provided insight into their cellular composition. These studies have demonstrated the presence of numerous cell types, including glial cells, retinal pigment epithelial cells, macrophages, and fibrocytes^2^. Notably absent from idiopathic ERMs are vascular channels. Consequently, ERM cells are strictly dependent on oxygen diffusion, presumably from the underlying inner retinal vasculature. This suggests that ERM cells survive – and proliferate – in an environment with a relatively low oxygen tension. Of note, in ischemic retinal disease, low oxygen tension leads to accumulation of the transcription factor, hypoxia-inducible factor (HIF)-1α, in many of the same cell types present in ERMs. Accumulation of HIF-1α in these cells leads to the secretion of vasoactive cytokines which promote vascular leakage and the development of ME^7–9^.

Interestingly, spectral domain optical coherence tomography (sdOCT) images from patients with visually-significant ERMs often demonstrate accumulation of interstitial fluid and increased central subfield thickness (CST) similar to those observed in patients with ME associated with ischemic retinal disease, such as diabetic macular edema, or cystoid macular edema in patients with retinal vein occlusions. A pathogenic link between ME associated with ERMs and that associated with retinal vascular disease is supported by immunohistochemical studies demonstrating expression of HIF-1α and the HIF-1-regulated gene product, vascular endothelial growth factor (VEGF), in surgically-removed idiopathic and diabetic ERMs^10–12^. Collectively, these studies implicate vasoactive and/or inflammatory cytokines in the promotion of vascular leakage and the development of ME in ERM patients.

While vitrectomy surgery and removal of the ERM remains the standard of care for visually-significant ERMs, the above observations have encouraged some clinicians to extend the use of pharmacologic therapies targeting VEGF (e.g., with anti-VEGF therapies) or inflammatory cytokines [e.g., with steroids or non-steroidal anti-inflammatory drugs (NSAIDs)] to treat ME in ERM patients. However, there are currently no clinical data to support – or refute – the use of pharmacologic therapies that target vasoactive and inflammatory mediators to treat ME in ERM patients. Although vitreous samples from patients with ERMs have been included in control groups in many prior studies examining the expression of inflammatory cytokines in patients with, for example, ischemic retinal disease, the relative levels of these cytokines in ERM patients compared to non-ERM controls, and the correlation of their concentration to the level of ME, has not previously been examined. Thus, it remains unknown whether vasoactive cytokines or inflammatory mediators are increased in ERM patients with ME and may therefore serve as therapeutic targets in its management.

In an effort to explore the potential value (or lack thereof) of non-surgical (pharmacologic) therapeutic options for symptomatic ERMs with ME, we examined the expression of vasoactive and inflammatory cytokines in the vitreous of patients with idiopathic ERMs compared to control patients. The results of this pre-clinical study could provide the foundation – or undermine the rationale – for a clinical trial assessing the use of specific pharmacologic therapies for the treatment of ME in ERM patients.

## Materials and Methods

### Ethics, consent and permissions

The research protocol was approved by the ethics review board of the Johns Hopkins School of Medicine. The study procedures were carried out in accordance with institutional guidelines and the Declaration of Helsinki. Signed informed consent was obtained from all patients after a full explanation of the procedures. All experiments were performed in accordance with relevant guidelines and regulations.

### Patient Selection and Samples

Institutional Review Board approval from the Johns Hopkins School of Medicine was obtained for all patient samples used in this study. Inclusion criteria included any patient undergoing vitrectomy for ERMs or for other causes. Exclusion criteria included ischemic retinal disease, uveitis, retinal detachment within 1 year of sample collection, or ME from another cause, and patients with a history of pre-operative anti-VEGF therapy, steroids or NSAIDs. Diabetic patients with active proliferative diabetic retinopathy were included as a positive control. Pure undiluted vitreous aspirates (approximately 1 mL) were collected from consenting patients at the Wilmer Eye Institute undergoing vitrectomy surgery. Consent was written and voluntary without stipend. Samples were immediately centrifuged at 16,000 × g for 5 minutes at 4 °C, and the aqueous component was stored at −80 °C prior to analysis.

### ELISA

Angiopoietin 2 (ANGPT2), Angiopoietin-like 4 (ANGPTL4), hepatocyte growth factor (HGF), monocyte chemoattractant protein-1 (MCP-1), pentraxin 3 (PTX3), and VEGF ELISA kits were purchased from R&D systems. Undiluted vitreous samples were analyzed for ANGPT2, ANGPTL4, MCP-1, PTX3 and VEGF, and 1:5 diluted (in PBS) vitreous samples were analyzed for HGF. ELISAs were performed according to the manufacturer’s protocols.

### Cytokine Array

Human Cytokine Array (ARY022, R&D systems) was performed according to the manufacturer’s protocols using 50 µL of undiluted pure vitreous samples. Densitometry of the array spots was performed using ImageJ software. The full list of cytokines used in this cytokine array is available at the manufacturer’s website (https://www.rndsystems.com/products/proteome-profiler-human-xl-cytokine-array-kit_ary022).

For the array studies, to minimize confounding variables that could influence the expression levels of cytokines detected by the array, we excluded patients with a history of any prior retinal surgery, ischemic retinal disease, neovascular AMD, uveitis, prior retinal detachment, or glaucoma. We also excluded patients with a history of intra- or periocular injections, or any recent history (within 1 month) of prescribed topical drops for any cause. We limited controls to patients without any history of posterior segment pathology (i.e., only patients who underwent vitrectomy for visually-significant vitreous opacities were include as controls). Using these strict inclusion criteria, we identified 16 ERM patients and 8 control patients for the array studies (Table 1).

**Table 1:** Patient samples used in cytokine array for potential identification of upregulated proteins in patients with epiretinal membranes who had mild, moderate, or severe ME compared to control patients.

Patient	Age	Sex	DM	CVD	Phakic Status^a^	Pre-Op OCT CST (μm)	Pre-Op VA	Pre-Op VA LogMAR	Post-Op OCT CST (μm)	Post-Op VA	Post-Op VA LogMAR
**Mild ME (250**–**349** **μm)**											
ERM 1	71	F	Yes	Yes	PP	266	20/160 − 1	0.923	223	20/50	0.398
ERM 2	70	M	No	Yes	PP	273	20/20 − 1	0.020	—	—	—
ERM 3	72	F	Yes	No	P	331	20/160 − 1	0.923	223	20/50	0.398
ERM 4	61	M	No	Yes	P	341	20/40	0.301	433	20/40	0.301
**Moderate ME (350–449 μm)**											
ERM 5	67	F	No	Yes	P	362	20/100	0.699	428	20/20	0.000
ERM 6	91	F	No	Yes	P	410	20/200	1.000	367	20/60	0.477
ERM 7	66	M	No	Yes	P	419	20/40	0.301	295	20/25 − 1	0.117
ERM 8	67	M	No	Yes	P	441	20/50–1	0.418	400	20/20	0.000
**Severe ME (≥450 μm)**											
ERM 9	59	M	No	No	PP	457	20/30	0.176	380	20/20	0.000
ERM 10	65	F	No	Yes	P	460	20/30	0.176	445	20/60	0.477
ERM 11	60	M	Yes	Yes	P	505	20/150	0.875	352	20/70	0.544
ERM 12	79	F	No	Yes	P	518	20/40 − 2	0.341	382	20/30 − 2	0.216
ERM 13	58	M	No	Yes	P	547	20/40 − 2	0.341	360	20/20	0.000
ERM 14	66	F	No	Yes	P	570	20/80	0.602	419	20/200	1.000
ERM 15	59	F	Yes	No	P	588	20/70 + 1	0.524	432	20/20	0.000
ERM 16	59	F	No	Yes	P	610	20/64	0.505	423	20/50	0.398
**Controls**											
Control 1	76	F	Yes	Yes	PP						
Control 2	86	M	No	Yes	PP						
Control 3	92	F	No	No	PP						
Control 4	60	F	No	Yes	P						
Control 5	71	M	No	Yes	PP						
Control 6	73	M	No	Yes	PP						
Control 7	70	M	No	Yes	PP						
Control 8	78	F	No	Yes	PP						

^(a)^Phakic status at the time of sample collection. DM, diabetes mellitus; CVD, cardiovascular disease; P, phakic; PP, pseudophakic; OCT, spectral domain optical coherence tomography; CST, central subfield thickness; VA, visual acuity. Control patents included only patients undergoing vitrectomy for visually-significant vitreous opacities without any history of posterior segment pathology.

### Statistical Analysis

For our power calculations, we estimated that the minimum biologically (and clinically) relevant increase of vasoactive or inflammatory cytokines in the vitreous of ERM patients, compared to control patients (without ERMs or ME from another cause), would be a 2-fold increase. By comparison, the vitreous levels of the vasoactive mediator VEGF in PDR patients is over 100-fold higher than in control patients. Our pilot studies demonstrated that vitreous concentration of VEGF is approximately 10 pg/mL in non-diabetic control patients. To detect a biologically relevant increase in the levels of VEGF in ERM patients with ME, we assigned μ1 = vitreous [VEGF] in ERM patients = 20 ng/ml; μ2 = vitreous [VEGF] in control patients = 10 ng/ml; and the common standard deviation for vitreous [VEGF] in control patients, Σ = 6. For α = 0.05 and power = 0.80, we calculated that we would need a minimum of 6 patients in each ERM sub-group to sufficiently power our study to observe a statistically significant, biologically relevant increase in vasoactive mediators or inflammatory cytokines in ERM patients with ME.

Results from clinical samples are shown as mean ± SD. Statistical differences between groups were determined by Student’s t-test, Wilcoxon signed-rank test, Mann-Whitney U test, Kruskal-Wallis test, and multiple regression analysis when indicated. Statistical analysis was performed using Microsoft Office, Prism 6.0 software (GraphPad), and Stata 13 (Stata Corp, College Station, Texas). *p < 0.05; **p < 0.01; ***p < 0.001; ****p < 0.0001.

## Results

### Peri-foveal vascular leakage in ERM patients who had severe ME

**Figure 1:**
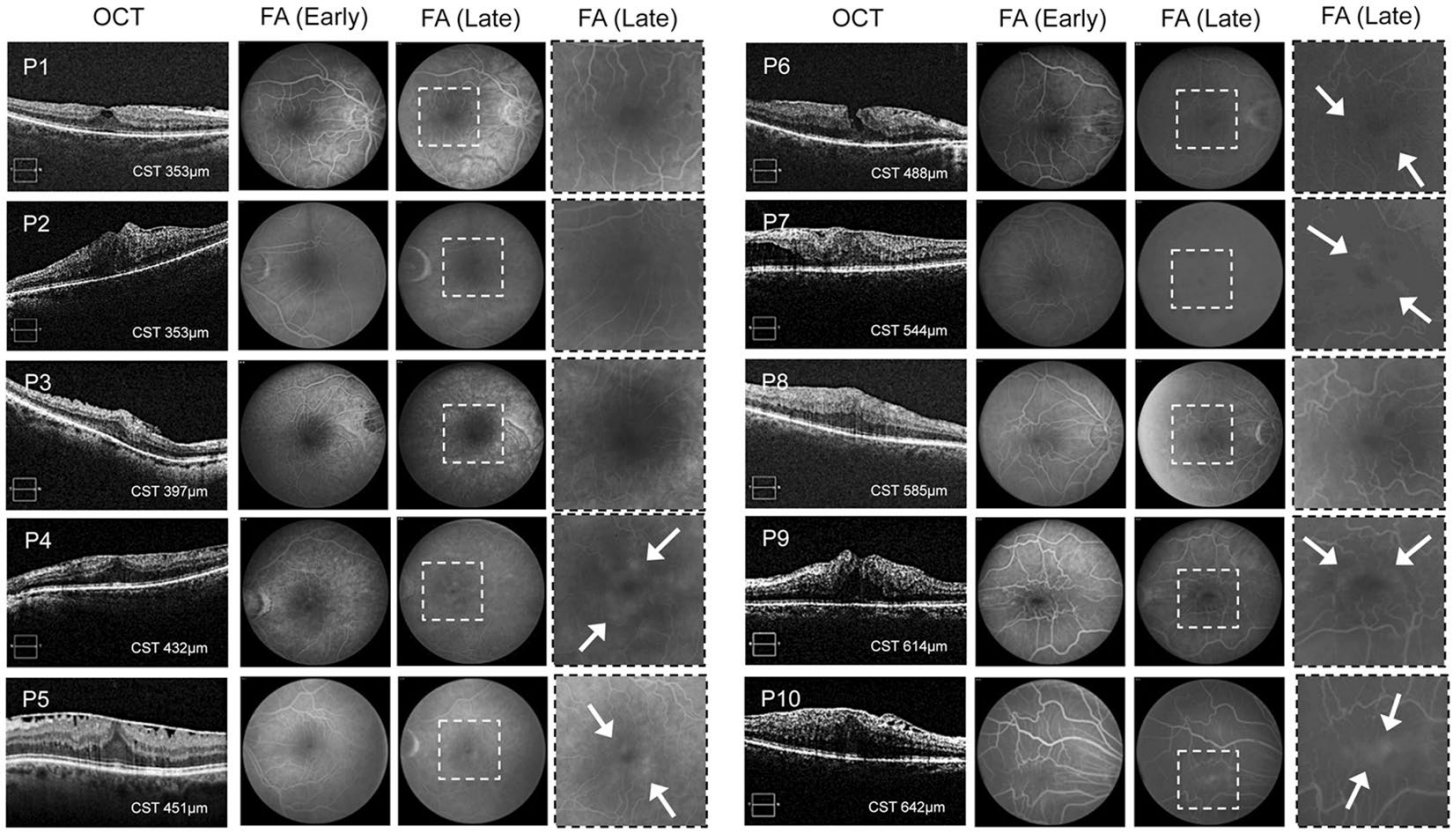
sdOCT and FA images from patients’ fovea-involving epiretinal membranes. Spectral domain optical coherence tomography (sdOCT) (*left*) demonstrates epiretinal membranes (ERMs) and interstitial fluid with increased central subfield thickness. Early and late fluorescein angiography (FA) images (*right*) demonstrate peri-foveal vascular leakage (arrows) in a subset of ERM patients (highlighted in inset).

To begin exploring the contribution of vasoactive mediators to vascular permeability and the development of ME in ERM patients, we initially performed a pilot prospective study assessing the presence or absence of fluorescein angiographic (FA) evidence of vascular leakage and sdOCT evidence of interstitial fluid in patients with ERMs. Patients with other causes of vascular leakage (e.g., ischemic retinal disease, uveitis, choroidal neovascular disease, post-surgical cystoid ME, etc.) were excluded. FA images demonstrated evidence of peri-foveal vascular leakage in a subset (6/10) of ERM patients (Fig. 1). Pre-operative sdOCT images were used to stratify ME severity by CST thickness (eTable 1), and FA images from these patients were examined for evidence of vascular leakage. In patients with “severe” ME (defined as CST > 450 μm), leakage on FA was observed in 5/6 patients, compared to 1/4 patients with a CST < 450 μm.

### Expression of classic vasoactive mediators in pure undiluted vitreous from ERM patients

The presence of peri-foveal vascular leakage in ERM patients who had severe ME supports a role for vasoactive cytokines in the pathogenesis of ME in ERM patients. We therefore performed a case-control study using quantitative enzyme-linked immunosorbent assays (ELISAs) to determine the vitreous concentrations of a prominent vasoactive cytokine, VEGF, in patients undergoing pars plana vitrectomy for ERMs (eTable 2) compared to control patients.

**Figure 2:**
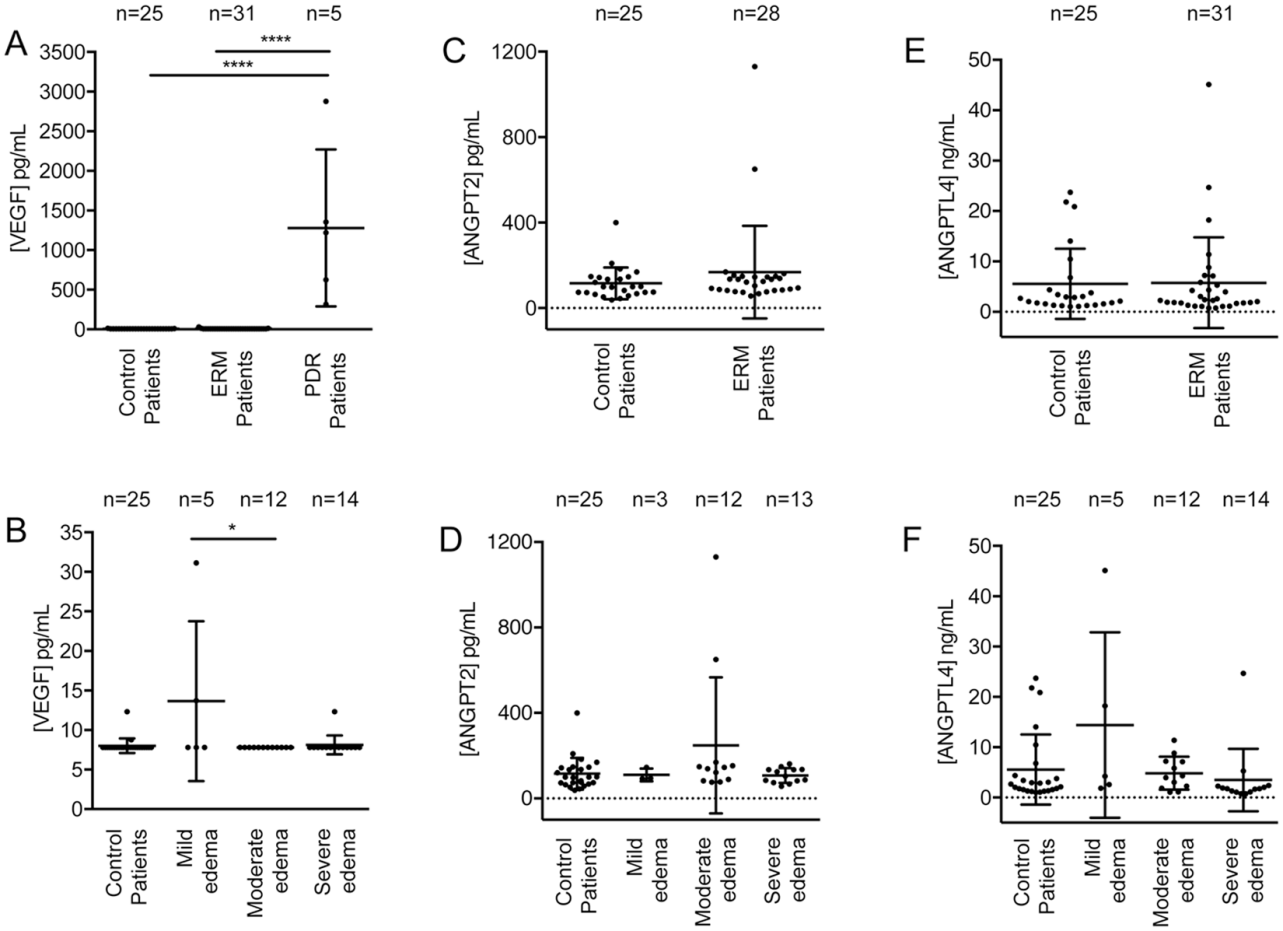
Vitreous concentrations of VEGF, ANGPT2 and ANGPTL4 in patients with ERM with ME and controls. (**A**, **C** and **E**) Vitreous vascular endothelial growth factor (VEGF) (**A**), angiopoietin-2 (ANGPT2) (**C**), and angiopoietin-like 4 (ANGPTL4) (**E**) in patients with epiretinal membrane (ERM) and macular edema (ME) compared to controls, and, for VEGF (**A**), ﻿also compare﻿d to patients with proliferative diabetic retinopathy. (**B, D** and **F**) Subgroup analysis of vitreous VEGF (**B**), ANGPT2 (**D**), and ANGPTL4 (**F**) concentrations in ERM patients who had mild (250–349 μm), moderate (350–449 μm), and severe ME (≥450 μm), compared to controls.

ERM patients demonstrated a significant improvement in their CST on post-operative sdOCT (eFigure 1A, *p* = 0.002) and a corresponding improvement in their post-operative visual acuity (eFigure 1B, *p* < 0.001). We found no significant increase in vitreous concentrations of VEGF in the ERM (n = 31) compared to control (n = 25) patients (eTable 3 and Fig. 2A, *p* = 0.72). In fact, vitreous VEGF levels in ERM patients were below the detectable level in 90% (28/31) of ERM patients. As a positive control, we examined VEGF levels in the vitreous of patients with active proliferative diabetic retinopathy (n = 5), and observed markedly elevated levels in 5/5 patients tested (Fig. 2A, *p* < 0.0001). To determine whether a subgroup of ERM patients had a subtle elevation of vitreous VEGF levels, we stratified ERM patients by their degree of ME as determined by CST on sdOCT. Patients were divided into “mild” ME (CST = 250–349 μm, a group in which we did not observe vascular leakage on FA); “moderate” ME (CST = 350–449 μm); and “severe” ME (CST ≥ 450 μm, a group in which we saw vascular leakage in the majority of patients on FA). Vitreous VEGF levels in each of the ERM subgroups did not significantly differ from control patients (Fig. 2B, *p* > 0.05). Multiple regression analysis showed that vitreous VEGF concentrations were not independently associated with CST in ERM patients with ME when controlling for age, sex, ethnicity, body-mass index (BMI), diabetes, cardiovascular disease and phakic status (results not shown).

To assess whether HIF-1-regulated vasoactive cytokines other than VEGF could be contributing to the development of ME in ERM patients (independent of, or in combination with low levels of VEGF), we next explored the abundance using ELISAs, of angiopoietin 2 (ANGPT2)^13^ and angiopoietin-like 4 (ANGPTL4)^14, 15^ in the vitreous of ERM (n = 28–31) patients compared to controls (n = 25). Both ANGPT2^13^ and ANGPTL4^14, 15^ have previously been implicated in the promotion of vascular permeability in ischemic retinal disease^14, 15^. Similar to the findings with VEGF, we found no significant increase in vitreous ANGPT2 or ANGPLT4 concentrations in ERM patients with ME when compared to control patients (Fig. 2C and E, *p* = 0.20 and 0.96, respectively). There were also no significant differences in the levels of ANGPT2 or ANGPTL4 in ERM patients who had mild, moderate, or severe ME when compared to control patients (Fig. 2D and F, *p* > 0.05). Multiple regression analysis showed that vitreous ANGPT2 concentrations were not independently associated with CST in ERM patients with ME when controlling for age, sex, ethnicity, body-mass index (BMI), diabetes, cardiovascular disease and phakic status (results not shown). However, initial multiple regression analysis suggested that vitreous ANGPTL4 concentrations from ERM patients with moderate (but not mild or severe) ME may be independently associated with CST (results not shown). Careful examination of the raw data revealed 3 extreme outliers (greater than 3 times the interquartile range) that inflated the mean. Subsequent multiple regression analyses performed after removal of these 3 outliers demonstrated that vitreous ANGPTL4 concentrations were not independently associated with CST in ERM patients with ME (regardless of the level of edema) when controlling for age, sex, ethnicity, body-mass index (BMI), diabetes, cardiovascular disease and phakic status (results not shown).

### Expression of inflammatory cytokines in pure undiluted vitreous from ERM patients

The above observations suggested that the HIF-1-regulated vasoactive cytokines that contribute to the development of ME in ischemic retinal disease and in choroidal neovascular disease are unlikely to contribute to the development of ME in ERM patients. Increased expression of inflammatory vasoactive cytokines has been implicated in the promotion of vascular permeability and the development of ME in patients with ischemic retinal disease and choroidal neovascular disease who are unresponsive to anti-VEGF therapy. Inflammation and increased expression of inflammatory cytokines have been hypothesized to contribute to the development of ERMs. We, therefore, next set out to examine whether non-classical vasoactive inflammatory cytokines may contribute to the promotion of ME in patients with ERMs. To this end, we took an unbiased approach and analyzed levels of 102 inflammatory mediators using a cytokine array. To minimize confounding variables that could influence the expression levels of cytokines using the array, we excluded patients with a history of any prior retinal surgery, ischemic retinal disease, neovascular AMD, uveitis, prior retinal detachment, glaucoma or a history of intra- or peri-ocular injections, or any recent history (within 1 month) of prescribed topical drops for any cause. Patients who underwent vitrectomy for visually-significant vitreous opacities were include as controls. Using these strict inclusion and exclusion criteria, we identified 16 ERM patients and 8 control patients (Table 1). The ERM group included patients who had mild (n = 4), moderate (n = 4), and severe (n = 8) ME, as defined above.

**Figure 3:**
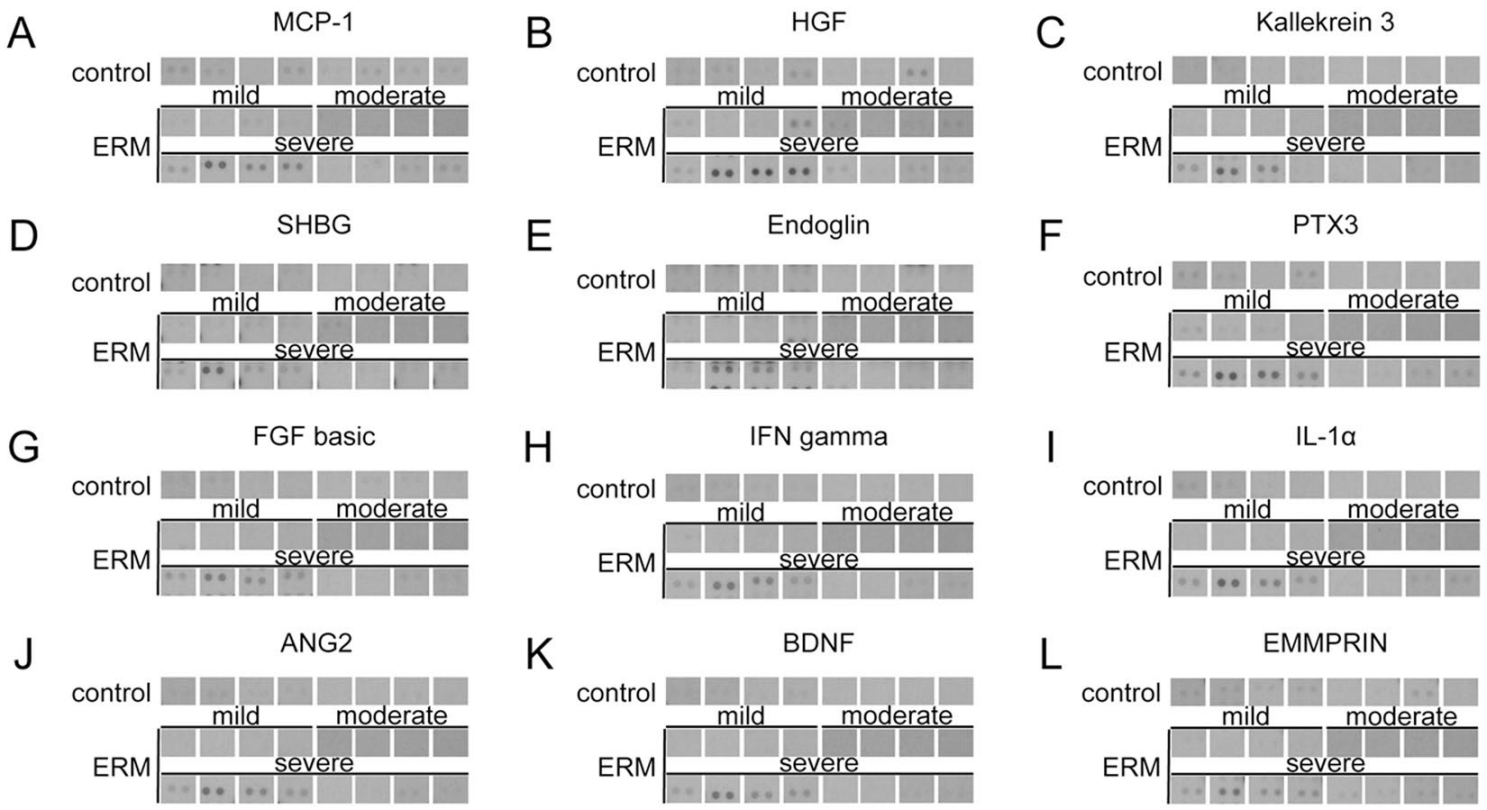
Cytokine array determined expression of non-classical vasoactive cytokines in epiretinal membrane (ERM) patients who had mild (250–349 μm), moderate (350–449 μm), and severe ME (≥450 μm) compared to control patients. (**A**) Monocyte chemotactic protein-1 (MCP-1), (**B**) hepatocyte growth factor (HGF), (**C**) kallekrein 3, (**D**) sex hormone-binding globulin (SHBG), (**E**) endoglin, (**F**) pentraxin 3 (PTX 3), (**G**) basic fibroblast growth factor (FGF basic), (**H**) interferon gamma (IFN gamma), (**I**) interleukin-1α (IL-1α), (**J**) angiopoietin 2 (ANG2), (**K**) brain-derived neurotrophic factor (BDNF), and (**L**) extracellular matrix metalloproteinase inducer (EMMPRIN) array expression in ERM patients who had mild (250–349 μm), moderate (350–449 μm), and severe ME (≥450 μm), compared to controls.

Using the array, we identified 4 cytokines on the array that were present at similar concentrations in all 24 vitreous samples (including all ERM patients and control paitents) tested (eFigure 2), and may serve the purpose of vitreous “housekeeping” cytokines when assessing cytokine expression using ELISAs. Increased levels of 12 cytokines were detected in the vitreous of a subset of patients with ERMs and severe ME (Fig. 3 and eTable 4). Among these 12 cytokines was angiopoietin 2 (ANGPT2), which was elevated in 3 of the 8 ERM patients who had severe ME (Fig. 3). However, as described above, an increase in ANGPT2 was not observed by quantitative ELISA analysis of a larger group of ERM patients compared to control patients (Fig. 2C and D). Interestingly, the 12 cytokines detected in ERM patients who had severe ME were all elevated in 3 particular ERM patients who had severe ME (eTable 5). Conversely, in 11 of the 16 ERM patients, including all 8 ERM patients with mild or moderate ME, and in all 8 control patients, expression of all 102 inflammatory cytokines was similar or identical. In addition, expression of 88% (90/102) of the inflammatory cytokines included on the array were similar or identical in all 16 ERM and 8 control patients examined.

### Quantitation of non-classical vasoactive cytokines in ERM patients

**Figure 4:**
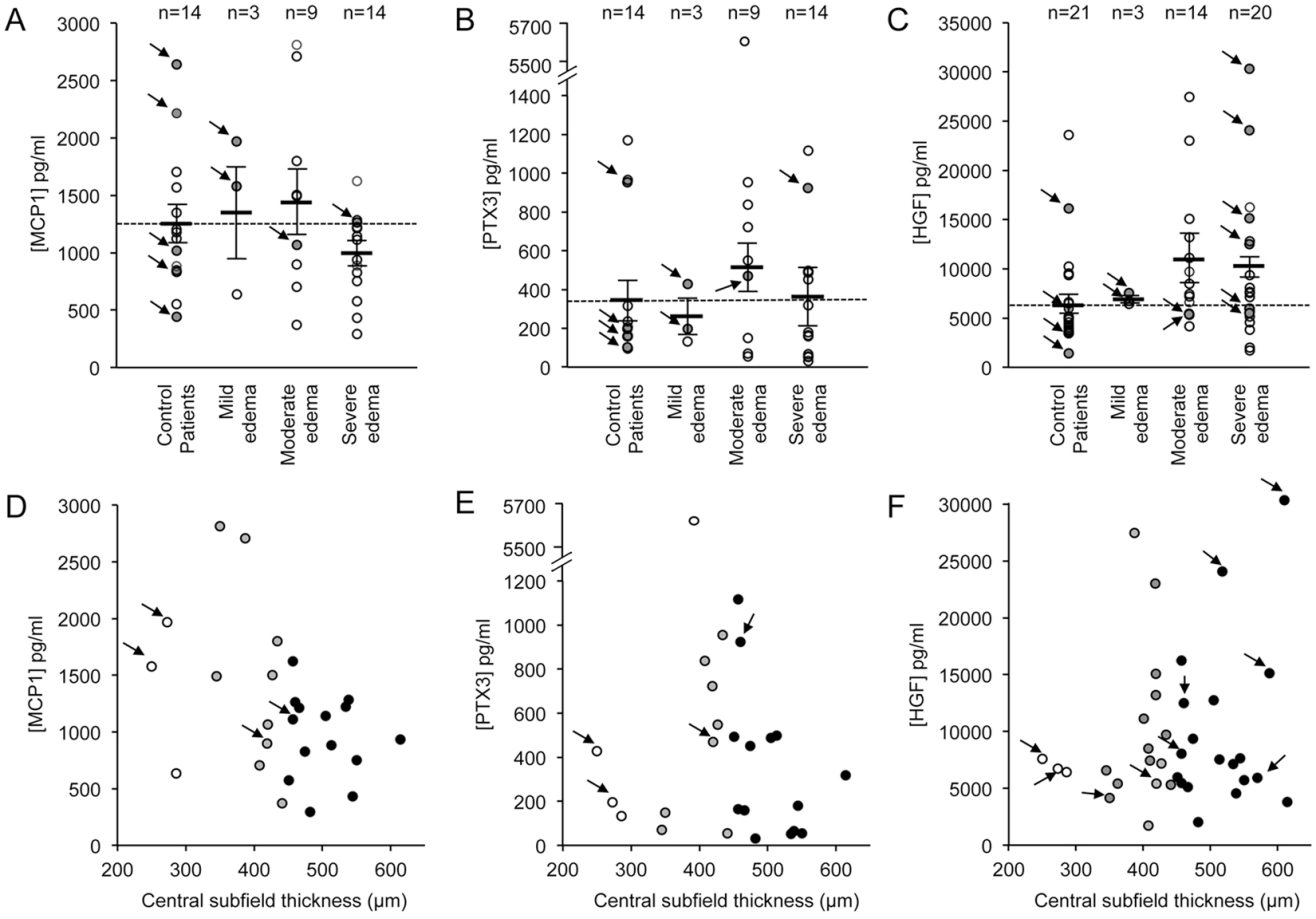
Vitreous concentrations of monocyte chemotactic protein-1 (MCP-1), pentraxin 3 (PTX 3) and hepatocyte growth factor (HGF) in epiretinal membrane (ERM) patients and controls by severity of ME and central subfield thickness. (**A**–**C**) MCP-1 (**A**), PTX3 (**B**), and HGF (**C**) expression in ERM patients who had mild (250–349 μm), moderate (350–449 μm), and severe ME (≥450 μm) compared to control patients. Dashed line is the average concentration for control patients and bold line is the average for each sub-group. (**D**–**F**) Vitreous concentrations of MCP-1 (**D**), PTX3 (**E**), and HGF (**F**) and their corresponding central subfield thickness in ERM patients. Empty circles represent patients who had mild ME, grey circles represent patients who had moderate ME, and solid circles represent patients who had severe ME. Arrows represent samples used for the analysis in the cytokine array in Fig. 3.

The incongruity between array and quantitative ELISA data for ANGPT2 prompted us to quantify in the vitreous the expression of candidate vasoactive cytokines that were identified in the array as elevated in ERM patients who had severe ME. We therefore examined the levels of the vasoactive inflammatory cytokines elevated in at least half (≥4/8) of the vitreous samples from ERM patients who had severe ME. This included monocyte chemoattractant protein-1 (MCP-1) and pentraxin-3 (PTX3), both of which were increased in 5 of the 8 ERM patients who had severe ME, as well as hepatocyte growth factor (HGF), which was increased in 4 of the 8 ERM patients who had severe ME. Although all three of these cytokines had previously been implicated in the promotion of vascular permeability^16–18^ and ME^19–23^ using ELISA, we did not observe a statistically significant difference in the vitreous levels of MCP-1, PTX3, or HGF between control patients and ERM patients who had mild, moderate, or severe ME (Fig. 4A, B and C).

For HGF, we did observe a modest increase in ERM patients who had moderate and severe ME, and this increase approached statistical significance for ERM patients who had moderate ME (*p* = 0.07). We therefore examined the correlation of HGF with the CST on sdOCT to assess further if HGF could contribute to the development of ME in ERM patients. Despite evidence of a modest (but not statistically significant) increase in HGF expression in ERM patients who had moderate and severe ME, we did not observe any correlation between a patient’s CST and their corresponding vitreous HGF levels (Fig. 4F). Similar non-correlative results were obtained for MCP-1 and PTX3 (Fig. 4D and E).

## Discussion

The possible benefit of medical management to treat ME in ERM patients includes delaying or avoiding intraocular surgery and its attendant risk of retinal detachment, infection, and loss of vision. This has encouraged some clinicians to consider extending the use of anti-VEGF therapy for the treatment of ME in ERM patients. We demonstrate here, however, that despite the similarities with ME due to ischemic retinal disease on sdOCT (e.g., accumulation of interstitial fluid in the inner retina) and FA (e.g., peri-foveal vascular leakage), and despite prior studies suggesting that HIF-1α and VEGF can be detected by non-quantitative immunohistochemical analyses in idiopathic ERMs^10–12^, VEGF levels in the vitreous of ERM patients are no higher than in control patients. Indeed, vitreous VEGF levels were undetectable in 90% of ERM patients tested. By contrast, vitreous levels of VEGF are markedly elevated (over 100-fold) in patients with proliferative diabetic retinopathy, a disease in which therapies targeting VEGF have proven effective. We further demonstrate that two other HIF-1-regulated vasoactive secreted proteins previously implicated in the promotion of vascular permeability in ischemic retinal disease, ANGPT2 and ANGPTL4, are also not significantly increased in patients with ERMs with associated ME compared to control patients. Collectively, these observations suggest that therapies targeting VEGF or other HIF-1-regulated vascular permeability factors may not affect ME associated with ERMs.

In patients with ischemic retinal diseases and ME who are unresponsive to anti-VEGF therapy, the use of steroids has shown some promise^24^. This observation supports the theory that inflammatory cytokines could function as non-classical vasoactive mediators and contribute to the development of ME, alone or in combination with VEGF. Inflammation and increased expression of inflammatory cytokines have been implicated in the development of ERMs^25^. This has motivated clinicians to try steroids and NSAIDs as adjuvant or mono-therapies for the treatment of ME associated with ERMs. However, using a cytokine array assessing the expression of 102 cytokines, many of which are reported to promote vascular permeability, we did not observe a marked difference in cytokine expression in the vitreous of most ERM patients with ME compared to control patients. Similarly, we did not observe a marked difference in the cytokine expression profile in the vitreous of ERM patients who had mild, moderate, or severe ME compared to one another, or compared to controls. These observations undermine the rationale for the use of anti-inflammatory agents for the treatment of ME in ERM patients. Indeed, expression of inflammatory cytokines was similar or identical in 11/16 ERM patients with ME and in 8/8 control patients. Also, expression of the 88% (90/102) of the inflammatory cytokines included on the array was similar or identical in all ERM and control patients examined. Collectively, these data suggest that while inflammatory cytokines could play a role in the early stages leading to the development of an ERM, they are unlikely to play a prominent role in the development of ME in ERM patients. These observations provide an explanation for the lack of efficacy observed in prior clinical studies assessing the use of adjuvant pre- or post-operative intraocular or systemic steroids to address ME in patients with ERMs despite surgical removal of the membranes^26–28^.

Conversely, in a subset of ERM patients who had severe ME, we did identify 12 candidate vasoactive cytokines with increased expression as compared to ERM patients who had mild or moderate ME, or control patients without ERMs. Interestingly, the 12 cytokines identified were all elevated in 3 of the 8 ERM patients who had severe ME. After a careful review of the medical records from these three patients, including an analysis of their demographic information, past medical histories, past ocular histories, and details obtained from their operative notes, no unifying factor could be identified that distinguished these 3 patients from the other 5 ERM patients who had severe ME, or from the 8 ERM patients who had mild or moderate ME, or the 8 control patients. These results suggested that there could be a subset of ERM patients who had severe ME for whom medical management using therapies targeting (specific) inflammatory mediators might be appropriate.

To characterize further the role of these inflammatory cytokines in ERM patients, we examined more closely the expression levels of the 3 cytokines (i.e., MCP-1, PTX3, and HGF) elevated on the array in at least half of the (≥4 of the 8) ERM patients who had severe ME. These cytokines were elevated in the original array study for the three patients described above as well as for at least one additional ERM patient who had severe ME. MCP-1 is expressed by fibroblasts and glial cells, which are also found in ERMs^29–31^, and has been implicated in the promotion of vascular permeability in the central nervous system^16^. More recently, expression of MCP-1 has been shown to be associated with ME in patients with uveitis, diabetic retinopathy, and exudative age-related macular degeneration^19–21^. Similarly, it has previously been reported that expression of PTX3 increases vascular permeability after ischemia-reperfusion injury^17^. Moreover, vitreous concentrations of PTX3 are increased in patients with branch retinal vein occlusions with associated ME, and correlate with increasing CST^22^. HGF and the HGF receptor (c-MET) have also been shown to increase retinal vascular permeability in a time- and dose-dependent manner^18, 23^.

Despite these promising supportive pre-clinical studies, quantitation of the levels of these three inflammatory cytokines by ELISA in a larger group of ERM patients failed to corroborate the array data. Expression of these cytokines was not increased in ERM patients with ME compared to control patients and did not correlate with the CST in patients with ERMs and moderate or severe ME. These results highlight the point that results from cytokine arrays must be corroborated by quantitative assays, and that ELISAs remain the gold standard to quantitate cytokine expression in vitreous samples.

Several other vasoactive cytokines, including basic fibroblast growth factor^32^, interferon-γ^33^, endoglin^34^, brain-derived neurotrophic factor^35, 36^, and interleukin-1α^37^, were all increased in the same 3 ERM patients who had severe ME described above. However, 4 (of 12) cytokines identified using the cytokine array were not increased when examined by quantitative ELISA in a larger group of ERM patients (compared to controls) and did not correlate with the level (or presence) of ME in ERM patients. This weakens enthusiasm for a causative role of one of these other vasoactive cytokines identified in the array in the development of ME in ERM patients.

Strengths of this study include the systematic qualitative assessment of vasoactive mediators in the vitreous of ERM patients with mild, moderate, or severe ME, compared to control patients, and the unbiased approach used to identify inflammatory cytokines that could also contribute to the promotion of ME in ERM patients. By challenging the results from the cytokine array using quantitative ELISAs, we demonstrated that there is little difference in the expression of vasoactive mediators and inflammatory cytokines in the vitreous of ERM patients compared to controls.

Limitations of this study include the study size. To calculate the sample size for our studies, we assumed the minimum biologically relevant increase of vasoactive or inflammatory cytokines in the vitreous of ERM patients compared to control patients would be a 2-fold increase (by comparison, PDR patients demonstrate a more than 100-fold increase in VEGF compared to non-diabetic controls). While the number of samples included in our study exceeded the sample size we calculated would be necessary to detect a statistically-significant biologically relevant increase in vasoactive and inflammatory cytokines in ERM patients with ME compared to control patients, we cannot rule out the possibility that a larger sample size may have identified a more subtle (i.e., less than 2-fold) increase in the vasoactive mediators examined; whether such a modest increase in vasoactive and inflammatory cytokines levels would be biologically relevant (and whether inhibiting such a minor increase would be clinically relevant) remains debatable.

Inclusion of additional samples in the cytokine array may have also resulted in the detection of other cytokines (albeit in a subset of ERM patients). Although unbiased, the cytokine array also limited us to the detection of only the 102 inflammatory cytokines included on the array. Thus, we cannot exclude the contribution of other (yet-to-be identified) vasoactive mediators and inflammatory cytokines not tested. We also cannot rule out the potential contribution of expression of vasoactive mediators or inflammatory cytokines as an aggregate. The combined effect of a modest elevation of multiple inflammatory cytokines and/or vasoactive mediators on the development of ME in patients with ERMs (or ME associated with other diseases) remains unexplored.

Despite these limitations, our study suggests that the risks of intravitreal injections (e.g., endophthalmitis and retinal tears/detachments) and the risks of intra- or periocular steroids (e.g., increased intraocular pressure and cataract formation) outweigh their potential benefits for ERM patients. Our results also do not provide support for the use of NSAIDs to treat ME in ERM patients. Although the risks associated with topical NSAIDs (e.g., corneal toxicity) may be small, the financial cost can be significant for patients. We conclude, instead, that mechanical traction and microvascular damage by the membrane remain the most likely causes for vascular leakage in ERM patients. Accordingly, surgical removal of the membrane is currently the most appropriate therapeutic approach to address the development of visually-significant ME in patients with ERMs.

## Electronic supplementary material

Supplementary Information
