# Supplementary material for: Lack of Evidence for Vasoactive and Inflammatory Mediators in the Promotion of Macular Edema Associated with Epiretinal Membranes
Source: Sci Rep. 2017 Sep 6;7:10608. doi: 10.1038/s41598-017-08997-6 (PMC5587698; doi:10.1038/s41598-017-08997-6)
Supplement: Supplementary file 1 — Supplementary Information [file 41598_2017_8997_MOESM1_ESM.pdf]

Lack of Evidence for Vasoactive and Inflammatory Mediators in the Promotion of Macular Edema Associated with Epiretinal Membranes

Brooks P. Applewhite, M.D.[1#], Savalan Babapoor-Farrokhran, M.D.[1#], David Poon, M.D.[1], Syed Junaid Hassan, M.S.[1], Elizabeth Wellmann, A.A.[1], Howard S. Ying, M.D., Ph.D.[2], Gregg L. Semenza M.D., Ph.D.[3], Silvia Montaner M.P.H., Ph.D.[4], and Akrit Sodhi M.D., Ph.D.[1]*

[#]B.P.A. and S.B.F. contributed equally to this manuscript.

[1]Wilmer Eye Institute, Johns Hopkins University School of Medicine, Baltimore, MD 21287, USA
[2]Department of Ophthalmology, Boston University School of Medicine, Boston, MA 02346, USA
[3]Vascular Program, Institute for Cell Engineering; Departments of Pediatrics, Medicine, Oncology, Radiation Oncology, Biological Chemistry, and Genetic Medicine, Johns Hopkins University School of Medicine, Baltimore, MD, 21205, USA
[4]Department of Oncology and Diagnostic Sciences, School of Dentistry; Department of Pathology, School of Medicine; Greenebaum Cancer Center, University of Maryland, Baltimore, MD 21201, USA

Running head: Vasoactive cytokines, Macular Edema & Epiretinal Membranes

*Correspondence:
Akrit Sodhi, M.D., Ph.D.
Wilmer Eye Institute, Johns Hopkins School of Medicine
400 N. Broadway St., Smith Building, 4039
Baltimore, MD 21287, United States
FAX: 410-614-8577
Email: asodhi1@jhmi.edu

<u>List of Supplement Elements:</u>

- **eTable 1**
- **eTable 2**
- **eFigure 1**
- **eTable 3**
- **eFigure 2**
- **eTable 4**
- **eTable 5**

eTable 1. Characteristics of patients with fovea-involving ERMs.

Patient	Age	Sex	Phakic Status[a]	OCT CST (μm)	VA	VA LogMAR
P1	59	F	P	353	20/20 -1	0.020
P2	70	F	P	353	20/50 -1	0.418
P3	79	F	PP	397	20/25 -2	0.137
P4	68	M	PP	432	20/40	0.301
P5	62	M	P	451	20/63 +2	0.458
P6	82	M	PP	488	20/50-1	0.418
P7	70	M	P	544	20/60	0.477
P8	72	M	P	585	20/80 +2	0.562
P9	68	F	PP	614	20/150	0.875
P10	78	F	P	642	20/30	1.176

(a) Phakic status at the time of sample collection. P, phakic; PP, pseudophakic; OCT, optical coherence tomography; CST, central subfield thickness; VA, visual acuity.

eTable 2. Pre- and post-operative characteristics of patients with idiopathic ERMs with ME used in analyses.

Patient	Phakic Status[a]	Pre-Op OCT CST (μm)	Pre-Op VA	Pre-Op VA LogMAR	Post-Op OCT CST (μm)	Post-Op VA	Post-Op VA LogMAR
ERM 1	PP	266	20/160 -1	0.923	223	20/50	0.398
ERM 2	PP	273	20/20 -1	0.020	--	--	--
ERM 3	P	331	20/160 -1	0.923	223	20/50	0.398
ERM 4	P	341	20/40	0.301	433	20/40	0.301
ERM 5	P	362	20/100	0.699	428	20/20	0.000
ERM 6	P	410	20/200	1.000	367	20/60	0.477
ERM 7	P	419	20/40	0.301	295	20/25 -1	0.117
ERM 8	P	441	20/50 -1	0.418	400	20/20	0.000
ERM 9	PP	457	20/30	0.176	380	20/20	0.000
ERM 10	P	460	20/30	0.176	445	20/60	0.477
ERM 11	P	505	20/150	0.875	352	20/70	0.544
ERM 12	P	518	20/40 -2	0.341	382	20/30 -2	0.216
ERM 13	P	547	20/40 -2	0.341	360	20/20	0.000
ERM 14	P	570	20/80	0.602	419	20/200	1.000
ERM 15	P	588	20/70 +1	0.524	432	20/20	0.000
ERM 16	P	610	20/64	0.505	423	20/50	0.398
ERM 17	PP	299	20/150	0.915	--	--	--
ERM 18	P	387	20/60 +2	0.437	284	20/40 -1	0.321
ERM 19	PP	402	20/50 -1	0.418	390	20/40	0.301
ERM 20	P	410	20/40 +3	0.241	331	20/25 -1	0.117
ERM 21	P	418	20/40 -2	0.341	368	20/20	0.000
ERM 22	PP	419	20/40	0.301	314	20/32	0.204
ERM 23	P	420	20/50 -2	0.438	351	20/25 +2	0.057
ERM 24	P	427	20/40	0.301	356	20/30	0.176
ERM 25	P	436	20/25	0.097	353	20/32 +2	0.164
ERM 26	P	457	20/60 +2	0.437	547	20/40 -1	0.321
ERM 27	P	482	20/32	0.224	491	20/50	0.398
ERM 28	PP	505	20/60	0.477	338	20/25 -2	0.137
ERM 29	P	514	20/32 -2	0.244	413	20/20	0.000
ERM 30	P	544	20/35 -2	0.283	406	20/20	0.000
ERM 31	PP	614	20/150	0.338	458	20/70	0.544

(a) Phakic status at the time of sample collection. P, phakic; PP, pseudophakic. OCT, optical coherence tomography; CST, central subfield thickness; VA, visual acuity. Mean time separating pre-op and post-op visual acuity and CST assessment is 164 days (range 21-400 days).

eFigure 1. CST and visual acuity outcomes for patients with ERM with ME following surgical intervention.

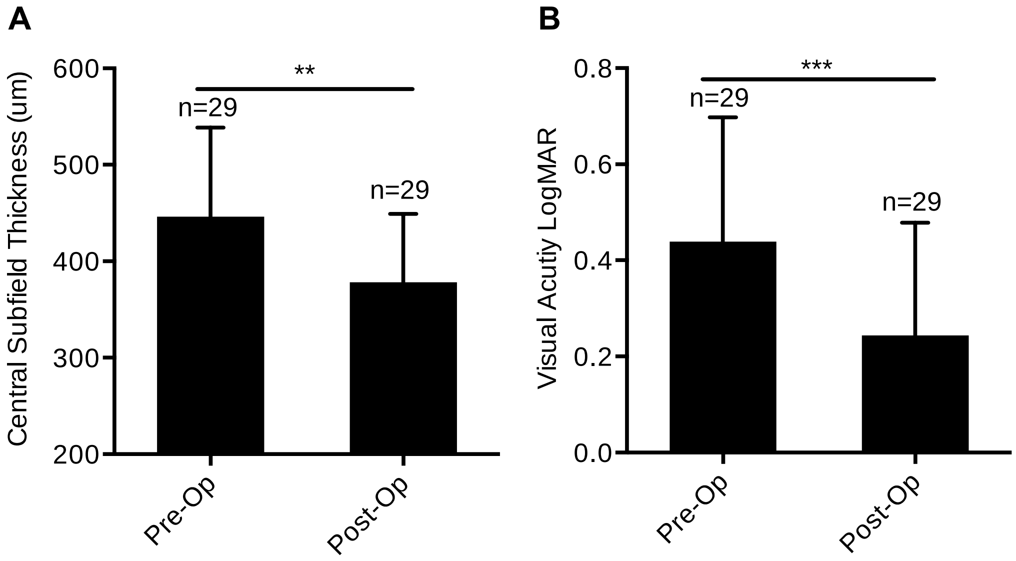

Surgical outcomes for idiopathic epiretinal membranes (ERMs) with ME as reflected by change in central subfield thickness (CST) (A) and visual acuity (B). Mean time separating pre-op and post-op visual acuity and CST assessment is 164 days (range 21-400 days).

eTable 3. Patient characteristics of vitreous samples analyzed with ELISA and cytokine array.

Patient	Age	Sex	Diabetes Mellitus
**ERM Patients**			
ERM 1	71	F	Yes
ERM 2	70	M	No
ERM 3	72	F	Yes
ERM 4	61	M	No
ERM 5	67	F	No
ERM 6	91	F	Yes
ERM 7	66	M	No
ERM 8	67	M	No
ERM 9	59	M	No
ERM 10	65	F	No
ERM 11	60	M	Yes
ERM 12	79	F	No
ERM 13	58	M	No
ERM 14	66	F	No
ERM 15	59	F	Yes
ERM 16	59	F	No
ERM 17	91	M	No
ERM 18	68	F	No
ERM 19	65	M	Yes
ERM 20	71	F	No
ERM 21	73	F	No
ERM 22	73	F	No
ERM 23	64	M	No
ERM 24	60	M	No
ERM 25	59	F	Yes
ERM 26	65	F	No
ERM 27	46	F	No
ERM 28	68	F	No
ERM 29	73	M	No
ERM 30	63	M	No
ERM 31	68	F	No
ERM 32	62	M	No
ERM 33	84	F	No
ERM 34	79	M	No

eTable 3. Patient characteristics of vitreous samples analyzed with ELISA and cytokine array (continued).

ERM 35	65	M	No
ERM 36	75	M	No
ERM 37	67	F	No
ERM 38	59	F	No
ERM 39	70	F	No
ERM 40	81	M	No
ERM 41	56	F	No
ERM 42	62	M	No
ERM 43	71	M	No
ERM 44	63	M	No
ERM 45	70	F	No
ERM 46	79	M	No
ERM 47	69	F	No
ERM 48	63	M	No
**Control Patients**			
Control 1	76	F	Yes
Control 2	86	M	No
Control 3	92	F	No
Control 4	60	F	No
Control 5	71	M	No
Control 6	73	M	No
Control 7	70	M	No
Control 8	78	F	No
Control 9	62	M	No
Control 10	64	M	No
Control 11	62	F	No
Control 12	43	F	No
Control 13	61	F	No
Control 14	65	F	No
Control 15	74	M	No
Control 16	84	F	No
Control 17	55	M	No
Control 18	46	M	No
Control 19	70	M	No
Control 20	74	F	No
Control 21	85	F	No

eTable 3. Patient characteristics of vitreous samples analyzed with ELISA and cytokine array (continued).

Control 22	74	M	No
Control 23	45	M	No
Control 24	82	M	No
Control 25	74	F	No
Control 26	64	M	No
Control 27	58	F	No
Control 28	51	M	No
Control 29	85	M	No
Control 30	52	F	No

eFigure 2. Cytokines in the array with similar concentrations in patients with ERM with ME and control patients.

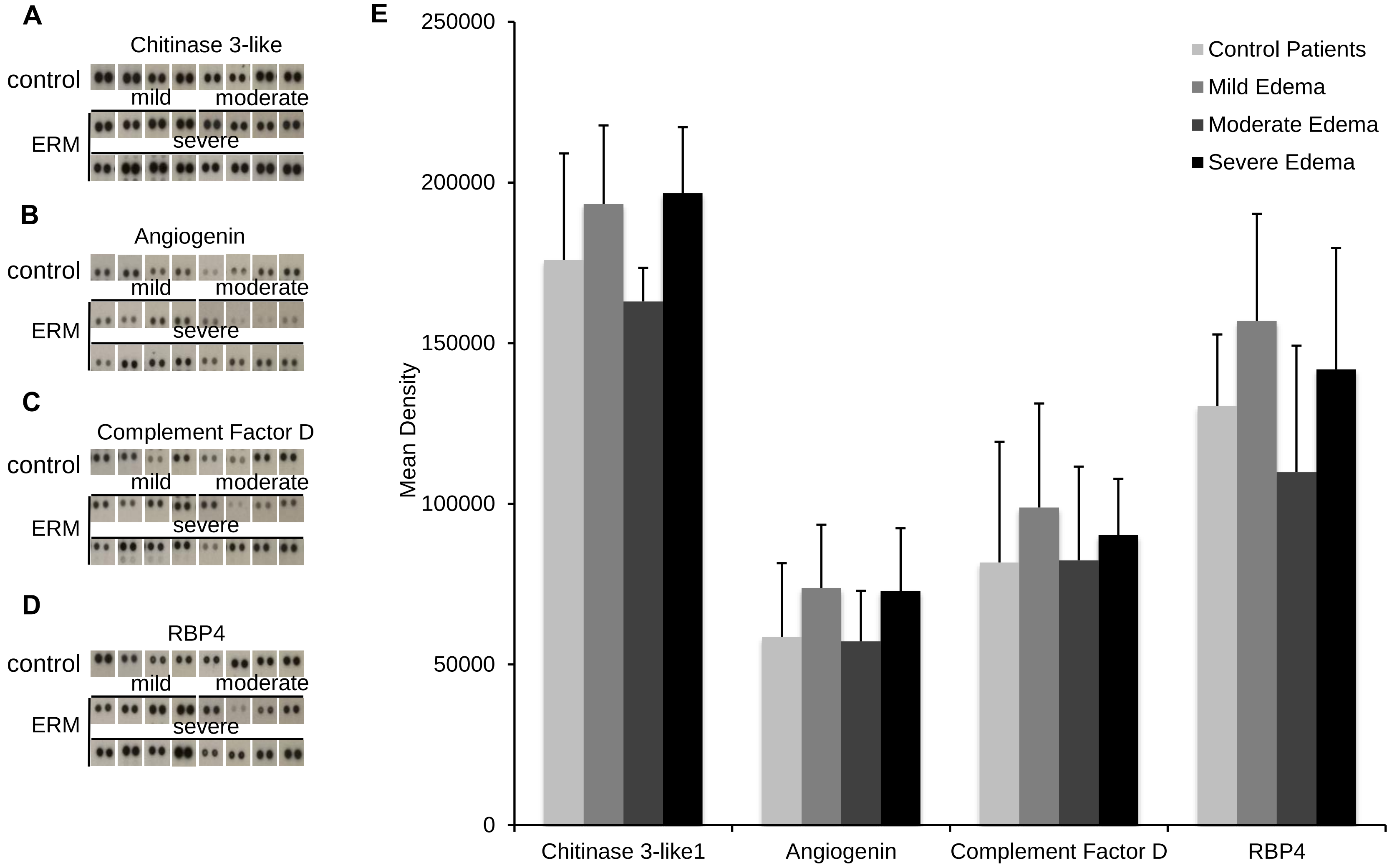

Cytokine arrays identified 4 out of 102 proteins (A) chitinase 3-like 1, (B) angiogenin, (C) complement factor D, and (D) retinol binding protein 4 (RBP4) that were present with similar concentrations amongst controls and patients with ERMs with mild, moderate, and severe ME. (E) Intensity analysis of dot blot showing mean density of spots from 4 cytokines. Densities are normalized to internal assay controls.

eTable 4. List of cytokines **identified in the cytokine array whose levels were increased in ERM patients with severe ME compared to ERM patients with mild or moderate ME and to control patients.**

Gene/Cytokine	Control (n=8)	Mild (n=4)	Moderate (n=4)	Severe (n=8)
CCL2/MCP-1	0	0	0	5
HGF	0	0	0	4
SHBG	0	0	0	3
ENG/Endoglin	0	0	0	3
FGF2/FGF basic	0	0	0	3
IFNG/IFN-gamma	0	0	0	3
ANGPT2	0	0	0	3
BDNF	0	0	0	3
IL1A/IL-1a	0	0	0	3
BSG/EMMPRIN	0	0	0	3
KLK3/Kallikrein 3	0	0	0	3
PTX3/Pentraxin 3	1	0	0	5

MCP-1: monocyte chemotactic protein-1; HGF: hepatocyte growth factor; SHBG: sex hormone binding globulin; FGF: fibroblast growth factor; IFN-gamma: interferon gamma; ANGPT2: angiopoietin 2; BDNF: brain-derived neurotrophic factor; IL-1α: interleukin-1α; EMMPRIN: extracellular matrix metalloproteinase inducer; PTX3: pentraxin 3.

eTable 5. List of ERM patients with severe ME with elevated levels of each of the cytokines identified in the cytokine array.

Gene/Cytokine[a]	ERM 9	ERM 10	ERM 11	ERM 12	ERM 13	ERM 14	ERM 15	ERM 16
CCL2/MCP-1		2.72	2.11	5.20	1.00	3.61		
HGF		55.00		112.30	1.00			30.20
SHBG		4.78		5.30	1.00			
ENG/Endoglin		1.23		6.44	1.00			
FGF2/FGF basic		0.31		3.57	1.00			
IFNG/IFN-gamma		1.49		6.28	1.00			
ANGPT2		2.96		9.05	1.00			
BDNF		2.47		6.61	1.00			
IL1A/IL-1a		1.60		2.25	1.00			
BSG/EMMPRIN		0.26		4.08	1.00			
KLK3/Kallikrein 3		1.00		2.45	1.00			
PTX3/Pentraxin 3		2.20	0.77	3.99	1.00	1.53		

(a) Values presented are fold-inductions normalized to patent ERM 13 who expressed the lowest recorded mean density for the majority of the listed cytokines among the subset of ERM patients with severe ME.

MCP-1: monocyte chemotactic protein-1; HGF: hepatocyte growth factor; SHBG: sex hormone binding globulin; FGF: fibroblast growth factor; IFN-gamma: interferon gamma; ANGPT2: angiopoietin 2; BDNF: brain-derived neurotrophic factor; IL-1α: interleukin-1α; EMMPRIN: extracellular matrix metalloproteinase inducer; PTX3: pentraxin 3.
